# The influence of inspiratory muscle training combined with the Pilates method on lung function in elderly women: A randomized controlled trial

**DOI:** 10.6061/clinics/2018/e356

**Published:** 2018-06-12

**Authors:** Guilherme Medeiros de Alvarenga, Simone Arando Charkovski, Larissa Kelin dos Santos, Mayara Alves Barbosa da Silva, Guilherme Oliveira Tomaz, Humberto Remigio Gamba

**Affiliations:** I Programa de Pos-Graduacao em Engenharia Eletrica e Informatica Industrial, Universidade Tecnologica Federal do Parana, Curitiba, PR, BR; II Departamento de Fisioterapia, Universidade Positivo, Curitiba, PR, BR

**Keywords:** Physical Therapy, Elderly, Inspiratory Muscle Training, Pulmonary Function

## Abstract

**OBJECTIVE:**

Aging is progressive, and its effects on the respiratory system include changes in the composition of the connective tissues of the lung that influence thoracic and lung compliance. The Powerbreathe® K5 is a device used for inspiratory muscle training with resistance adapted to the level of the inspiratory muscles to be trained. The Pilates method promotes muscle rebalancing exercises that emphasize the powerhouse. The aim of this study was to evaluate the influence of inspiratory muscle training combined with the Pilates method on lung function in elderly women.

**METHODS:**

The participants were aged sixty years or older, were active women with no recent fractures, and were not gait device users. They were randomly divided into a Pilates with inspiratory training group (n=11), a Pilates group (n=11) and a control group (n=9). Spirometry, manovacuometry, a six-minute walk test, an abdominal curl-up test, and pulmonary variables were assessed before and after twenty intervention sessions.

**RESULTS:**

The intervention led to an increase in maximal inspiratory muscle strength and pressure and power pulmonary variables (*p*<0.0001), maximal expiratory muscle strength (*p*<0.0014), six-minute walk test performance (*p*<0.01), and abdominal curl-up test performance (*p*<0.00001). The control group showed no differences in the analyzed variables (*p*>0.05).

**CONCLUSION:**

The results of this study suggest inspiratory muscle training associated with the Pilates method provides an improvement in the lung function and physical conditioning of elderly patients.

## INTRODUCTION

The aging population is prominent and growing exponentially in several countries and regions. Between 2015 and 2030, the number of people aged 60 and older will increase 56%, from 901 million to 1.4 billion, and by the year 2050, the world will have close to 2.1 billion elderly people 1.

A decrease in the strength of the respiratory muscles causes impairments in the performance of functional activities 2, such as routine activities, with varying degrees of complexity and energy costs. In addition, changes can be observed in the amount and composition of lung tissue components such as elastin, collagen and proteoglycans, which lead to a reduced chest compliance and increased pulmonary compliance 3.

In this sense, inspiratory muscle training appears to be a possible intervention that can minimize harmful effects on respiratory muscles in the elderly 4.

The Pilates method is used in clinical practice to promote muscle rebalancing with exercises that emphasize the powerhouse, also called the center of the body 5.

Based on this information, the aim of the present study was to analyze the influence of inspiratory muscle training associated with the Pilates method on the pulmonary function and aerobic capacity of the elderly.

## METHODS

### Study design

This was a randomized controlled trial wherein simple random sampling was performed; this method is based on the sequential selection of each sample unit with equal probability so that each unit has the same chance of being chosen 6.

The Research Ethics Committee of Universidade Positivo (Positivo University), under reference no. 1.114.692/2015, approved the study. After a detailed explanation of the aims, benefits and risks involved in this investigation, all the participants provided informed consent.

### Inclusion and exclusion criteria

This study included female participants with a minimum age of 60 years, who had no fractures in the 3 months prior to the study, did not use walking devices, and, if hypertensive, had a borderline hypertension of 140/90 mm/Hg. The excluded participants were those who had neurological lesions, were in the inflammatory phase of arthritis, practiced regular physical activity, and had less than an 85% attendance rate at the sessions. The initial analysis of the level of physical activity was conducted using the International Questionnaire of Physical Activity (IPAQ), and it showed that 100% of the elderly women were classified as active 7.

### Participants

Sampling was performed using the GPower 3.1.3 (Heinrich Heine University, Düsseldorf, Germany) application 8 with the following parameters: assuming tests with F family distribution, three groups, two dependent measures, an average effect size (0.3), a type I error of 0.05, a power equal to 0.84, and a total sample of 36 elderly women.

**Figure 1:**
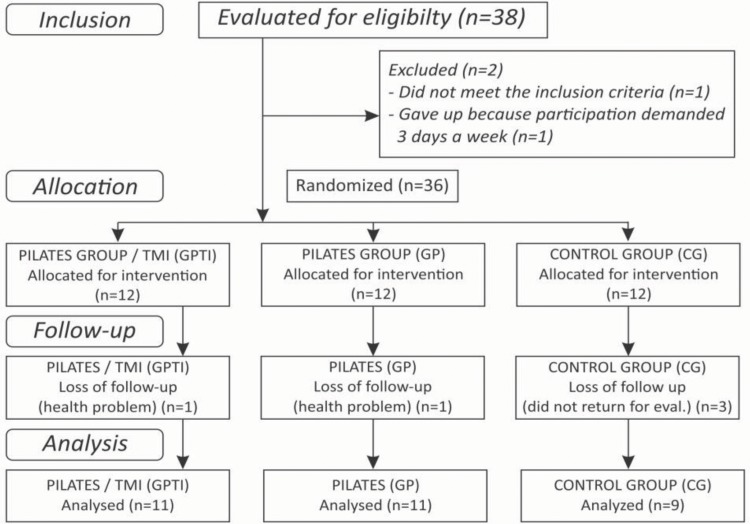
Flow Diagram

The study consisted of 12 volunteers in the group with the inspiratory muscle training and Pilates method intervention (GPTI), 12 volunteers in the Pilates intervention only group (GP), and 12 volunteers in the control group (CG), who did not undergo any intervention (Figure 1).

### Variable measurements

The evaluative sessions pre- and post-intervention, detailed below, comprised the following assessments of pulmonary function: forced vital capacity (FVC) and forced expiratory volume in first second (FEV1) 9. The spirometer used was a Contec® SP10 (Contec Medical Systems Co., Qinhuangdao, China). Respiratory muscle strength was assessed by determining the maximum inspiratory pressure (MIP) and maximum expiratory pressure (MEP) 10. The analog manovacuometer used was from Murenas® (Murenas Produtos para Saude Ltda., Juiz de Fora, Brasil). Pulmonary pressure (cm/H_2_O), power (watts), flow (liters/sec) and energy (joules). Powerbreathe® K5 (POWERbreathe International Ltd., England, United Kingdom) devices were used. In addition, a six-minute walk test (6MWT), an objective evaluation of aerobic capacity, was performed on a 20-meter runway 11, and an abdominal curl-up test was performed to measure the strength and resistance of the abdominal muscles based on the number of curl-up repetitions completed in 1 minute 12.

### Intervention

The study was conducted at the Physiotherapy Clinic of the Universidade Positivo (Positivo University) in Curitiba-Paraná. The intervention was performed 2 times a week, for a total of 20 sessions of inspiratory muscle training and Pilates method training that were 45 minutes long each.

### Inspiratory muscle training

For the GPTI, inspiratory muscle training was conducted using Powerbreathe® K5 devices, and the participants were instructed to perform 30 inspiratory efforts in 2 sets, with a 1-minute interval between sets. An initial load of 50% of the MIP of each individual was established, and it was increased by 10% every two weeks and assessed following the Pilates exercises.

### Pilates method

The following Pilates devices were used in this study: Cadillac, Combo Chair and Reformer. A total of 9 exercises were used per session, with 1 to 3 sets of 12 repetitions of each exercise, for a total duration of 45 minutes. The strength of the springs was adjusted individually and progressively according to the capacity of each participant and his/her ability to carry out the proposed exercises.

### Statistical analysis

The FVC, FEV1, MIP, MEP, 6MWT and abdominal curl-up data were evaluated for residual distribution patterns by the Shapiro-Wilk test and for homoscedasticity by the Bartlett test. Once the data were totally or partially in agreement with such assumptions, repeated-measures analysis of variance (ANOVA) was applied, followed by Tukey's N-HSD follow-up test. The pressure, power, flow and volume data after the use of inspiratory muscular training (IMT) in the GPTI were also evaluated for the assumptions of normality and homoscedasticity and were later evaluated by repeated-measures ANOVA.

For all analyses, the statistical software STATISTICA 7 13 was used, with a significance level of 0.05.

## RESULTS

### Participants

The mean age of the patients for each group was GPTI, 65.36 (±4.46); GP, 65.45 (±3.27); and CG, 73.33 (±5.55).

Table 1 shows the main findings. The protocol led to an increase in maximal inspiratory muscle strength (*p*<0.0001), maximal expiratory muscle strength (*p*<0.0014), 6MWT performance (*p*<0.01) and abdominal curl-up test performance (*p*<0.00001) (Table 1).

The spirometric values did not show significant differences; however, this test was only performed to rule out any ventilatory disturbances that could have affected the performance of the proposed exercises.

Table 2 shows the main findings. The protocol led to an increase in pressure and power pulmonary (*p*<0.0001) (Table 2).

## DISCUSSION

To our knowledge, no other randomized controlled trial has been published that assesses IMT associated with the Pilates method in the elderly population. A study by Lopes et al. 3 applied Pilates in the elderly and evaluated respiratory muscle strength but did not include randomization, IMT or a CG. In addition, a study by Souza et al. 4 analyzed the effect of IMT on respiratory muscle strength in the elderly but did not compare other exercise methods, as in the present study.

Studies report that Pilates in the elderly promotes improvements in static and dynamic balance, which minimizes the number of falls, as well as improving overall muscle strength, increasing flexibility in the lower limbs, improving quality of life, and increasing physical fitness and autonomy, thus corroborating the data from the present study 5,14,15.

One important result from the present study was the finding regarding MIP. After the intervention, both the group that received the intervention using the Pilates method and the group that received the intervention associated with respiratory muscle training showed significantly higher MIP values than did the control group (Table 1). There were significant intra- and intergroups gains in variables that were not seen in the CG.

Most previous studies have assessed IMT in groups of people with many different diseases, for example, the systematic review by Medeiros et al. (16), which reviewed IMT and respiratory exercises articles and included populations with chronic renal disease; these populations were not considered as active as the elderly women in our study.

Additionally, due to the absence of studies with a similar methodology as that used in our research, we were forced to compare our results with the research by Drăgoi et al. 17, which involved patients with ankylosing spondylitis and IMT; the characteristics of decreased mobility in the thoracic spine and ribs and consequent associated respiratory muscle weakness in young patients with ankylosing spondylitis are biomechanically comparable to those of the aged population.

Lopes et al. 3 conducted a clinical trial using Pilates in elderly women with a focus on respiratory muscle strength, applying their treatment in 22 sessions over 11 weeks; although they did not specify whether Pilates was on the ground or in an apparatus, they only used 5 exercises and found no significant gains in MIP. In our study, with a smaller number of sessions and a greater number of exercises, the MIP significantly evolved in the elderly. We highlight the use of the Powerbreathe® K5 device, which further favored the gain in this variable.

Ferreira et al. 18, in a study using Threshold® devices for six weeks of IMT, found a statistically significant improvement in MIP and MEP in hypertensive elderly women, which corroborates our results, although the elderly women in our study were considered active. Reychler et al. 19 worked with an IMT group using Threshold® devices for 15 minutes, 5 times a week. The group showed an average of 255 breaths per session. At the end of the 20 sessions, there was an improvement of 38% in MIP in the 16 patients. Buuren et al. 20 used pre-operative and post-operative muscle strength exercises in cardiovascular patients. During the pre-operative period, IMT can increase MIP and thus improve respiratory status, as well as improve maximal inspiratory force. Thus, the benefit of gain training, in cm/H2O, is approximately twice as high for weaker patients.

Additionally, in a program involving 30 visits using Threshold® devices, with 5 sets of 9 repetitions, 5 times a week, and an average of 225 breaths per week, an increase of 41.8% in MIP was observed 21. In addition, our results showed that the use of the Powerbreathe® K5 promoted an improvement of 68% in MIP with only 60 breaths per session in a total of 28 sessions. This finding shows greater gains with a much lower number of breaths.

Studies on exercise practice report that when given the correct stimulus, peripheral muscle strength can be improved at any age and that physical exercise is beneficial for respiratory muscles 3.

The improvements in MIP can be attributed to a strengthening of the inspiratory muscles, since the skeletal muscles are sensitive to adequate training programs, which corroborates the findings of Ferreira et al. 18.

Two systematic review studies found that in all articles included in the meta-analyses, IMT led to a significative improvement in MIP compared with that in all CGs in patients with congestive heart failure 22,23, thus corroborating the findings of the present study.

The abdominal muscles are the primary motor control in the MEP (Table 1), which evaluates the strength of the expiratory musculature. Effective expiration facilitates the elimination of CO_2_, gas exchanges, better ventilation and the oxygenation of cellular tissues, which are crucial for healthy aging 24.

It is worth noting that there was a significant increase in MEP and abdominal strength (Table 1), with increases in the number of repetitions in the abdominal curl-up test (124% in GPTI, 145% in GP and 5% in CG) that match a previous study 25; that study, in its systematic review of 133 articles, analyzed aspects related to the importance of central stabilization in the Pilates method and concluded that this combination helps the individual obtain strength gains, neuromuscular control, and muscle power and endurance, thus facilitating the balanced functioning of the muscles. The Pilates training group in the current study demonstrated equal improvements in the performance of the abdominal curl-up test, which shows that all the patients who underwent a Pilates method intervention effectively understood its principles.

A study that used Pilates with 33-year-old women twice a week for 12 weeks and assessed respiratory muscle function and strength found that there was no favorable significant differences in these variables between the Pilates group and the control group. These findings do not align with the results of our study, which showed that all the variables were significantly better in the intervention groups than in the CG, thus strengthening the importance of the association between IMT and Pilates 26.

Previous studies have shown that in the elderly population, the decrease in skeletal muscle mass and function is constant 27. In people over 65, this percentage reaches a 14% loss. The diaphragm is the main inspiratory muscle, responsible for 75% of inspiration 28. An equal amount of maintenance and gain becomes essential. Thus, our results are relevant, as these interventions can help avoid the risk of mortality, especially related to the susceptibility to lung diseases, which are the third leading cause of death in the elderly in Brazil 29,30.

Based on this information, the association between IMT and Pilates was the driving force for the present study. At the present juncture of population aging, an increase and association between methods and resources is necessary to avoid complications and reduce the mortality of this population.

Regarding physical conditioning, the 6MWT was used to measure the aerobic capacity of the elderly. All the elderly women in the intervention groups improved the distance traveled significantly (Table 1), corroborating previous studies by 31 and 32 where elderly patients performed exercises, demonstrating that exercise increases and maintains function and activity in the elderly 33.

In relation to the effects on the pulmonary variables of pressure, power, flow and energy, this protocol and training resource proved to be effective, increasing pulmonary volume, work and balancing energy expenditures (Table 2). Thus, new studies should take advantage of the variables provided by the equipment used in this research and consider that addressing questions in regard to respiratory capacity is not limited to the use of manovacuometry (MIP and MEP) 16,34–36.

We recognize the limitations of our study. The use of a fourth group, with the same characteristics of our sample, that exclusively used the IMT device would help analyze the exclusive influence of IMT in this population and determine if the device leads to similar benefits.

Further studies should be performed that analyze the respiratory variables of pressure, power, flow, and energy measured in this study, to compare our results and validate our methodology.

In conclusion, physiotherapy is an excellent ally in the prevention, promotion, and maintenance of health, quality of life and functional capacity in the gerontological population. The use of the Pilates Studio method, associated with technological equipment that allows a more detailed analysis and treatment of pulmonary conditions, strength, function and mobility, was shown to be beneficial for this type of application.

## AUTHOR CONTRIBUTIONS

Alvarenga GM and Gamba HR conceived and designed the study. Alvarenga GM, Charkovski SA, Santos LK, Silva MB and Tomaz GO were responsible for the acquisition, analysis and interpretation of the data. Gamba HR was responsible for administrative support. Alvarenga GM, Charkovski SA and Gamba HR were responsible for the critical revision of the manuscript for important intellectual content. All the authors have read and approved the final version of this manuscript.

## Figures and Tables

**Table 1:** Pre- and post-intervention pulmonary function comparisons between the GPTI (n=11), the GP (n=11) and the CG (n=9).

		Pre		Post		*p*-value
Parameter	Groups	Mean	SD	Mean	SD	
MIP (cm/H_2_O)	GPTI	36.55bA	8.86	78.55aA	14.12	<0.0001
	GP	51.64bA	17.93	66.18aA	15.73	
	CG	40.44aA	19.33	34.67aB	12.00	
MEP (cm/H_2_O)	GPTI	53.45aA	10.47	82.18bC	18.71	<0.0014
	GP	58.54aA	21.33	80.00bBC	16.78	
	CG	52.44aA	19.94	50.67aAB	17.09	
6MWT (meters)	GPTI	423.36aA	46.26	513.09aA	48.04	<0.01
	GP	406.54aA	100.32	515.18aA	117.04	
	CG	361.78aA	76.59	395.89aA	87.86	
ABD (repetitions)	GPTI	16.18aA	7.19	36.18aA	6.95	<0.00001
	GP	14.18aA	10.12	34.73aA	10.61	
	CG	13.11aA	10.00	12.44aA	11.94	

GPTI, inspiratory muscle training and Pilates method group; GP, Pilates group; CG, control group; MIP, maximal inspiratory pressure; MEP, maximal expiratory pressure; 6MWT, six-minute walk test; ABD, abdominal curl up.

Different lowercase letters indicate statistically significant differences intra-groups, *p*<0.05 (pre x post).

Different uppercase letters indicate statistically significant difference between groups, *p*<0.05.

**Table 2:** Pulmonary capacity results on the 1^st^, 14^th^ and 28^th^ days of intervention in the GPTI (n=11).

	Day 1		Day 14		Day 28		
Parameter	Mean	SD	Mean	SD	Mean	SD	*p*-value
Pressure (cm/H_2_O)	12.28	3.07	15.23	3.94	20.7	5.03	<0.0001
Power (watts)	1.8	1.16	3.49	1.4	4.56	1.43	<0.0001
Flow (liters/sec)	1.4	0.73	2.3	1.01	2.29	0.82	0.0009
Energy (joules)	57.74	26	59.57	16.12	62.5	22.57	0.7597

GPTI, inspiratory muscle training and Pilates method group.
